# Port Site Morbidities Following the Extraction of the Gallbladder from the Umbilical Port in Comparison to the Epigastric Port in Laparoscopic Cholecystectomy: A Double-Blinded, Randomized Controlled Trial

**DOI:** 10.7759/cureus.45770

**Published:** 2023-09-22

**Authors:** Atul Anand, Ashesh k Jha, Manoj Kumar, Subhash Kumar, Pragya Kumar

**Affiliations:** 1 General Surgery, All India Institute of Medical Sciences, Patna, IND; 2 Radiology, All India Institute of Medical Sciences, Patna, IND; 3 Community Medicine, All India Institute of Medical Sciences, Patna, IND

**Keywords:** port site pain, epigastric port, umbilical port, cholecystectomy, symptomatic gallstones

## Abstract

Background

Port site morbidities after laparoscopic cholecystectomy may be related to the port used for the extraction of the gallbladder. Prior randomized trials that tried to address the suitable port for gallbladder extraction showed mixed results favouring epigastric, whereas others favoured umbilical. Thus, the present study was conducted with the aim of finding a suitable port for gallbladder extraction after laparoscopic cholecystectomy.

Methodology

A total of 104 patients undergoing laparoscopic cholecystectomy were randomized to either the epigastric (Group 1) or umbilical (Group 2) port group for gallbladder extraction. Post-operative pain (by visual analogue scale (VAS)), the frequency of surgical site infection (SSI), and port site herniation were compared.

Results

Post-operative pain was lower in the umbilical port group in the initial 24 hours. The SSIs and port site herniation rates were lower in the umbilical port group; however, they were statistically not significant.

Conclusion

Less post-operative pain at the umbilical port may help with the early discharge of patients. In contrast to other studies, our trial had fewer infections and hernias in the umbilical port group.

## Introduction

The prevalence of gallstone disease (GSD) has been estimated to be approximately 4.15% (95% CI, 0.00-8.76) in the populations of the Gangetic Plains of northern India. [1] Laparoscopic cholecystectomy remains the treatment of choice for symptomatic gallstones. [2] The laparoscopic technique is associated with minor pain and early post-operative recovery. [3,4] However, port site morbidities such as post-operative pain, port site infection, and port site herniation can occur after laparoscopic cholecystectomy. There is a relative paucity of well-designed randomized controlled trials to evaluate the preferred port site for gallbladder extraction following laparoscopic cholecystectomy.

Both the umbilical and epigastric ports are used for gallbladder retrieval, depending on the surgeon's preference or local institutional guidelines. Various randomized controlled trials [5-12] have compared umbilical and epigastric sites for gallbladder extraction with mixed results, with some favouring umbilical and others epigastric port sites. Meta-analyses conducted by Sood et al. [13], Mongelli et al. [14], Kulkarni et al. [15], and Hajibandeh et al. [6] pointed out the heterogeneity of these individual trials and hence, the difficulty in deriving a concrete conclusion. Despite an extensive literature review, we could not find a single study employing both double-blinding and allocation concealment for gallbladder extraction via umbilical or epigastric port after laparoscopic cholecystectomy. Additionally, most of these trials did not mention the power of the study or the sample size calculation, and most studies have a follow-up period of only 24 hours.

These limitations prompted us to conduct a randomized trial by reworking the shortfall of prior studies to determine the preferred port site for gallbladder extraction.

## Materials and methods

This was a double-blinded, randomized-controlled superiority trial carried out at the All India Institute of Medical Sciences, Patna, Bihar, one of the largest tertiary care institutes in eastern India. After getting approval from the Institutional Ethics Committee (IEC), the trial was registered with the Central Trial Registry of India (CTRI) with registration number CTRI/2021/10/037123. The study duration extended from October 2021 to October 2022.

Consenting patients aged 18-80 years, belonging to the American Society of Anaesthesiologists (ASA) grades 1 and 2, undergoing an elective standard four-port laparoscopic cholecystectomy, were included in the study. Patients with acute cholecystitis, a body mass index (BMI) of more than 30 kg/m^2^, diabetes, the largest stone size of more than 2 cm in the pre-operative ultrasound scan, and/or bile spillage during surgery were excluded from the study. Patients with a history of previous surgery with an umbilical scar, prior episodes of biliary pancreatitis, and choledocholithiasis (post-endoscopic clearance) were not included.

The sample size was calculated based on the randomized controlled trial by Siddiqui et al. [5] In their study, the mean visual analogue scale (VAS) for pain at 12 hours after laparoscopic cholecystectomy in the epigastric group was 3.9±0.85, and that in the umbilical group was 2.4±0.79. Assuming a true difference in means of VAS score between the umbilical and epigastric port groups of -1.5 (i.e., 2.4-3.9) units and a pooled standard deviation of 0.82 units, the study required sample size of 47 for each group (i.e., a total sample size of 94, assuming equal group sizes) to achieve a power of 90% and a level of significance of 5% for declaring that the umbilical port is superior to the epigastric port for gallbladder extraction by changing the VAS score by minus one unit's margin of superiority (assuming that a smaller mean is desirable). Furthermore, assuming a 10% dropout rate from the study, the final sample size came out to be 104, with 52 patients in each arm. Although the minimum sample size was 104, 152 sealed envelopes were created with a block size of four, considering that after randomization, approximately 50 more patients may get excluded.

We used the sequentially numbered, opaque, sealed envelope (SNOSE) technique for allocation concealment. All numbers generated after randomization were placed in opaque envelopes by an independent observer. These opaque, sealed envelopes were opened by the nursing staff, who were not involved in data collection or analysis. Based on the random numbers retrieved upon opening the envelope, patients were allocated to either the epigastric or umbilical extraction groups. Both the patient and the follow-up surgeon were blinded to the intervention.

All laparoscopic cholecystectomies were performed by an experienced surgeon who had performed at least 30 laparoscopic cholecystectomies. The gallbladder was extracted via either the epigastric or umbilical ports. We have uniformly used 10 mm metallic ports at both epigastric and umbilical locations in all patients, i.e., similar port sizes at both locations were essential to preserve blinding. The follow-up surgeon was not present in the operation theatre at the time of the surgery and did not have access to the operating notes. We did not use endo-bags for gallbladder extractions as they were not readily available at our setup. Port sites were not infiltrated with local anaesthetic agents so as to quantify the actual pain, which was uninfluenced by local anaesthetics. Post-operative analgesia was standardized in both groups in the form of an injection of diclofenac sodium (1-2 mg/kg intravenously every 12 hours). Patients who still had severe pain despite the injection of diclofenac, that is, a VAS of more than or equal to seven, were given additional analgesia in the form of an injection of tramadol 1-2 mg/kg every four to six hours.

The same follow-up surgeon (who was blinded to the intervention) assessed pain, infection, and herniation throughout the study period. Post-operative pain at port sites was assessed in all patients by asking the severity of pain in their own language based on the VAS at three hours, six hours, nine hours, 12 hours, 24 hours, 48 hours, seven days, and 30 days after surgery by a blinded observer. To define port site infection, the CDC's definition of surgical site infection (SSI) was used by the attending surgeon.

The port sites were evaluated during the follow-up visits at seven and 30 days for SSI. Those with persistent SSI for 30 days were counted twice, i.e., on the seventh and on the 30th day.

Port site herniation was defined as either the presence of visible swelling at the port sites with cough impulse and/or reducibility, up at one and three months postoperatively or the presence of herniation reported by a senior radiologist using ultrasonography at three months. [16] The time for gallbladder extraction was measured by an independent person using a stopwatch. It measured the time period from dissecting the gallbladder from the liver bed until its actual extraction from the abdominal cavity.

Statistical analyses were performed using Jamovi software version 2.3.18 (The Jamovi Project (2023), retrieved from https://www.jamovi.org). Student’s t-test (or Welch’s t-statistics, in case the assumption of homogeneity of variances was not met) was used for continuous variables, and the chi-square test was used for categorical variables. A repeated measures analysis of variance (ANOVA) test was applied to compare VAS scores across different time points. Statistical significance was set at p<0.05.

## Results

**Figure 1:**
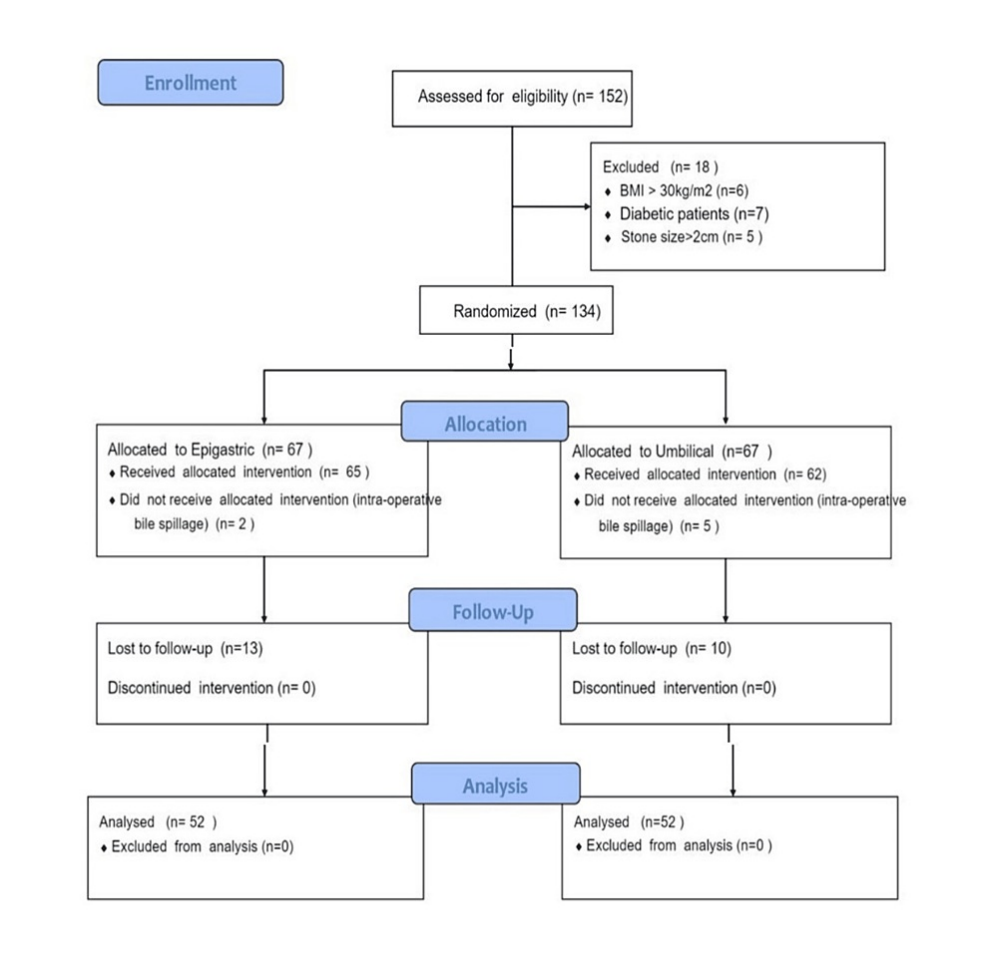
CONSORT diagram CONSORT: Consolidated Standard of Reporting Trials

Figure 1 shows the Consolidated Standard of Reporting Trials (CONSORT) diagram for the study.

Of 152 patients, 18 were excluded at baseline according to various exclusion criteria, as mentioned in the CONSORT diagram.

Therefore, 67 patients were assigned to the epigastric and umbilical port groups. Patients who had intra-operative bile spillage and those lost to follow-up were also excluded, resulting in 52 patients in each arm for analysis. Baseline variables (gender distribution, age, and BMI) were comparable in the two groups (Table 1).

**Table 1:** Baseline variables in the two groups

Variables	Group 1 (Epigastric group) n=52	Group 2 (Umbilical group) n=52	p-value
Females frequency (%)	36 (69.23%)	39 (75%)	p=0.512
Males frequency (%)	16 (30.77%)	13 (25%)	
Age (years) (mean ± S.D)	33.9±9.87	36.7±11.94	p= 0.188
BMI (kg/m^2^) (mean ± S.D)	23.5±3.75	23.8±4.23	p=0.762

**Figure 2:**
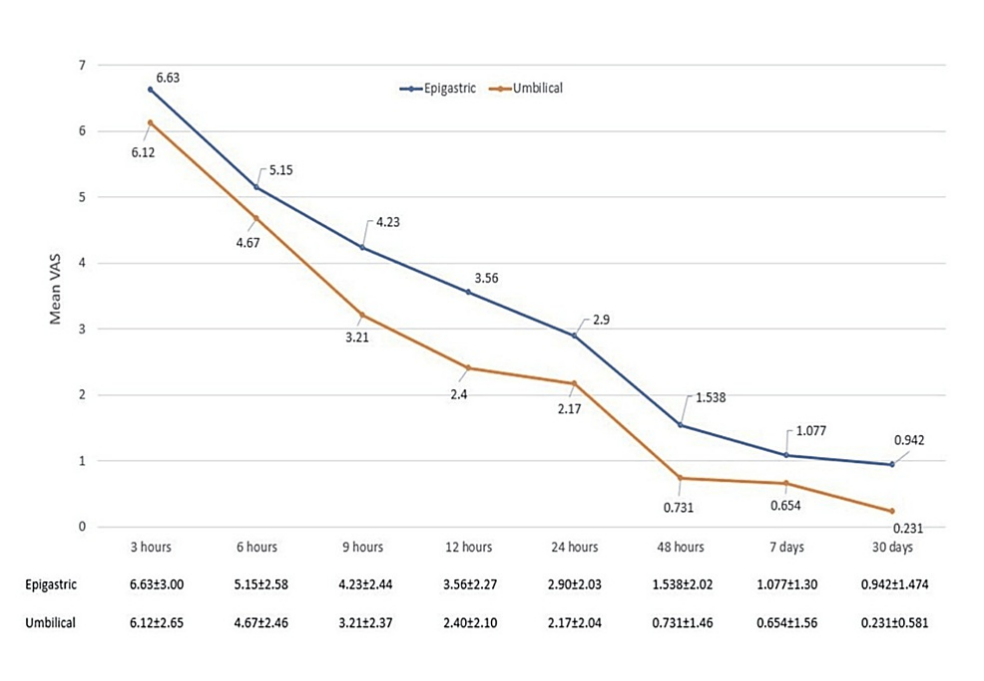
Line diagram showing variation in the mean pain score (±SD) by VAS with time VAS: visual analogue score

Figure 2 shows the mean pain scores (SD) by VAS in the two groups at specified time points.

We found that umbilical port extraction of the gallbladder led to decreased post-operative pain at all time points. However, the minimum time for this decline to be statistically significant was nine hours post-operatively. Thus, as per our study, although there was a reduction in post-operative pain in the umbilical port extraction group, a statistically significant difference was noted only at nine hours. In the post hoc analysis, when taking time as one factor and the port used for gallbladder extraction as the other factor in a two-factorial repeated-measures ANOVA, the mean difference in pain scores increased statistically significantly until 24 hours, with an increasing trend in the mean difference in both ports across most time frames. After 24 hours, the mean difference between the two port groups did not vary significantly (p > 0.05). The incidence of surgical site infection at seven days and 30 days post-operatively, the incidence of port site hernia at one month and three months post-operatively, and the intra-operative time for gallbladder extraction did not vary significantly in the two groups (Table 2).

**Table 2:** Comparison of surgical site infection (SSI), port-site hernia, and gallbladder extraction time in the two groups

	Group 1 (Epigastric group) n=52	Group 2 (Umbilical group) n=52	p-value
SSI at 7 days, frequency (%)	9 (17.3%)	6 (11.5%)	0.402
SSI at 30 days, frequency (%)	16 (30.8%)	9 (17.3%)	0.108
Hernia at 30 days, frequency (%)	0 (0)	0 (0)	
Hernia at 3 months, frequency (%)	6 (11.5%)	5 (9.6%)	0.750
Time (in seconds) for GB extraction (mean ± S.D)	226±111	249±125	0.315

## Discussion

The laparoscopic approach is preferred over the open technique for cholecystectomy in terms of length of hospital stay, costs, and overall quality of life. However, the preferred port site for gallbladder extraction remains a matter of debate amongst surgeons across the globe.

As reported by Rosero et al. [17], post-operative pain is among the top 10 reasons for hospital readmissions within 30 days of ambulatory laparoscopic cholecystectomy. Post-operative pain was responsible for hospital readmissions in 11.8% of patients, followed by infection in 10.4% of cases. Understandably, significant post-operative pain can impede early discharge after laparoscopic cholecystectomy; however, its significance is scarcely discussed in the literature. This could be due to the fact that pain following laparoscopic cholecystectomy is multifactorial and depends on a number of factors, including the degree of dissection in the liver bed, intra-operative peritoneal stretching, pressure [18], flow rate [19], nature [20], and the amount of residual gas. [21] In addition, the port site used for extraction can also influence the severity of pain at port sites.

Several studies have attempted to address the ports that should be used routinely for gallbladder extraction. However, most of these studies have a short follow-up duration of 24 hours. Most randomized trials do not report adequate sample size calculations, the power of the study, the randomization method, blinding, or allocation concealment. We attempted to limit these drawbacks in the present study to minimize selection and observer bias.

The only study that we could find that reported the methods of sample size calculation was by Siddique et al. [5] and Jain et al. [11]. Our sample size was calculated considering it to be a superiority trial, as previous studies reported mixed results, most favouring umbilical port sites and a few favouring epigastric port sites. On a similar note, with a power of 90% in the sample size calculation, the probability of making a type 2 error by wrongly failing to reject the null hypothesis decreases.

This study had robust inclusion and exclusion criteria. None of the previous studies have commented on the ASA status of the patients. Only patients with ASA grades one and two were included in our study. Patients with a BMI higher than 30 kg/m2 were excluded. In addition, patients with immunocompromised status, diabetic patients, and patients with acute cholecystitis were also excluded. Patients with intra-operative bile spillage were also excluded from the analysis.

This was necessary because higher ASA grades [22-24], emergency procedures [25], known diabetes status [25, 25-28], acute cholecystitis [22, 25-28], and intra-operative bile spillage are established risk factors for increased SSI incidence. [25] Only two previous studies [7, 10] excluded patients with a higher BMI. Their cut-off for exclusion was much higher (BMI> 40 kg/m2) than that used in our study (BMI > 30 kg/m2). As the latest World Health Organization (WHO) classification [29] considers a BMI of 30 kg/m2 as obesity class I, we decided to lower the cut-off BMI to exclude obese patients. Studies have also found that BMI > 30 kg/m2 is a risk factor for the development of surgical site infection after laparoscopic cholecystectomy. [30] In some randomized trials [5, 10], when extraction of the gallbladder from the abdominal cavity was difficult, the port sites were dilated. We believe that such practices may lead to increased pain scores and herniation rates. In this study, none of the ports were dilated in order to facilitate the delivery of gallbladder specimens via epigastric or umbilical port sites.

Females constituted nearly 75% of the study population. Many previous studies have found that female sex is a risk factor for the development of gallstone disease. [31-33] The mean age of patients in our study was 33.9±9.87 years in the epigastric arm and 36.7±11.94 years in the umbilical arm. In terms of the mean age, our randomized trial closely resembled that of Hajong et al. [9]. In their study, the mean age in the two arms was 33.48±10.6 years and 31.10±7.8 years, respectively. Most other trials [5,7,10] had a mean patient age of more than 40 years. Therefore, the patients in our study were younger than those in previous studies.

We followed the patient for post-operative port site pain at three-hour intervals initially for the first 12 hours. Hajong et al. [9] followed up with patients at one, six, 12, and 24 hours after surgery, whereas Siddiqui et al. [5] and Jain et al. [11] followed up with patients at one, six, 12, 24, and 36 hours after surgery. We feel that the patient is still under the effects of anaesthetic agents when pain is assessed as early as an hour after surgery. In contrast, Bashir et al. [7] and Shakya et al. [8] directly assessed patients at 24 hours and did not study immediate post-operative pain. Only Kaya et al. [10] studied the long-term effects of port site selection by assessing postoperative pain at the end of one month. We attempted to address this limitation by studying both immediate and delayed postoperative pain scores by following up with the patients at three-hour intervals initially, then at 24 hours, 48 hours, seven days, and finally on post-operative day 30.

Most previous randomized trials [5, 8, 9] found a statistically significant reduction in pain in the group in which the gallbladder was extracted via the umbilical port at all time points. We also found that umbilical port extraction of the gallbladder led to decreased post-operative pain at all time points. However, the minimum time for this decline to be statistically significant was nine hours post-operatively. The significant decline in pain scores before 24 hours may help discharge patients early, realizing the day-care concept behind laparoscopic cholecystectomy. The possible explanation could be due to fascial laxity at the umbilicus in comparison to the epigastric region, thereby requiring less stretching of fibres during umbilical port extraction.

In accordance with the recommendations of the European Society of Regional Anaesthesia and Pain Therapy (ESRA) guidelines [34], we used nonsteroidal anti-inflammatory agents for post-operative analgesia in both groups, dosed according to body weight as described in the methodology section above.

Because pain is a dynamic phenomenon, we studied the decline in pain scores at different intervals, considering the port factor (Figure 2), using a repeated-measures ANOVA test. None of the earlier studies have used this test to study the decline in pain scores. The difference in pain reduction after surgery was more marked for the first 24 hours in both groups, after which the two groups did not vary significantly in pain reduction. This means that regardless of the port used for gallbladder extraction, the patient will have a noticeable decrease in pain for the initial 24 hours only, after which both groups will experience a comparable decline.

Our superiority trial was designed with a power of 90%. During the sample size calculation, we assumed that umbilical port extraction of the gallbladder would decrease post-operative pain by at least one unit at 12 hours post-operatively. The final results showed a mean pain score of 3.56±2.27 in the epigastric port group and 2.40±2.10 in the umbilical port group at 12 hours (p = 0.008) suggesting that we met this primary objective.

The CDC classifies SSI into three types: superficial incisional SSI, deep incisional SSI, and organ/space SSI, depending on the depth of tissue involved [35]. We have limited our study to superficial incisional SSI. Although we excluded most of the patients with high-risk features, the incidence of port site infections in our study was much higher than others (14.4% at the end of one week and 24% at the end of a month after the procedure). Shakya et al. [8] had 4% SSI rates; Kaya et al. [10] reported 1.6%. Moreover, the incidence of SSI on post-operative day 30 is higher as compared to that on post-operative day seven in our study (an increase of 10%). None of the authors in the past have compared the incidence of SSI at two different time frames and thus failed to comment on the change in SSI rates over time.

The higher incidence of port site infections in our study may be partly explained by the type of population, mostly low- to middle-class families with overcrowded homes and poor sanitation. Though this should have necessitated the use of end bags during gallbladder extraction, they are costly and not readily accessible to most of our patients who belong to lower socio-economic strata. Nevertheless, this study served to highlight the increased infection rates post-laparoscopic cholecystectomy in our institution, mandating the usage of disposable ports thereafter.

Consistent with other randomized trials [8, 11, 12], our study showed a higher incidence of port site infections in the epigastric group. The umbilical port is usually visualized as a "dirty" port, considering its ignorance during bathing. However, we were unable to reach this conclusion in our study.

The umbilicus is usually considered a common site for acquired hernias in adults. Trocar site incisional hernia is an unusual complication after laparoscopic cholecystectomy, the incidence of which is reported to be approximately 1%-1.8% [36,37]. Our study failed to reach statistically significant results in terms of port site hernias. The incidence of port site hernia was almost comparable between the two arms in the present study: 11.5% in the epigastric port group and 9.6% in the umbilical port group. We routinely close the sheath at the umbilical port with polyglactin sutures; such a practice is not used at the epigastric port, which may explain the slightly increased hernia rates in the epigastric port group in our study, along with the increased incidence of SSI (a predisposing factor to hernia development) at the epigastric port. This finding was consistent with the results of a meta-analysis conducted by Francesco Mongelli et al. [14]. In an analysis of seven studies with a total of 876 patients, with the gallbladder being extracted via the epigastric port in 441 and umbilical port in 435 patients, the occurrence of trocar site hernias demonstrated an overall risk difference of 0.033 (favouring the epigastric trocar site and a 95% CI = 0.205-0.139, p = 0.706), which showed no difference between the two trocar sites. Similarly, a meta-analysis by Sood et al. [13] failed to show a statistically significant difference in trocar site hernias between the two groups. In contrast, the meta-analysis by Kulkarni et al. [15] clearly favoured epigastric port location for decreased hernia rates. In their study, the relative risk of developing a trocar site hernia was higher in the umbilical group arm (relative risk (RR) of 2.68; 95% CI, 1.06-6.80, p-value 0.04).

Another disadvantage of umbilical port extraction is the increased extraction time, as it requires repositioning the camera port prior to gallbladder extraction. We believe that the time from the dissection of the gallbladder off the liver bed until its actual extraction from the abdominal cavity accounted for the extra time consumed in repositioning the camera from the umbilical port to the epigastric port. Thus, we specifically measured this time. Siddiqui et al. [5] measured the overall time for surgery in both arms from incision to skin closure. The mean operative time was thus higher in their study at 52.5±12.1 minutes and 56.7±13.8 minutes (p-value of 0.078) in the epigastric and umbilical groups, respectively. Others, such as Bashir et al. [7], have specifically mentioned the time from the completion of the dissection of Calot's triangle and the clipping and cutting of the cystic duct and artery until their actual extraction from the abdominal cavity. In their study, the mean time was 10.62±4.611 and 8.64±4.182 in the epigastric and umbilical groups, respectively (p-value = 0.032, significant). Although we also found increased time spent repositioning the camera port (similar to Siddiqui et al. [5] and Sood et al. [13]), this did not translate into a statistically significant delay.

One of the major limitations of our study was its single-centre design. Further, multi-centre studies are required to compare these two ports. The maximum follow-up period in our study was three months, which may not be long enough to comment on port site herniation rates.

## Conclusions

In conclusion, the umbilical port site appears to have a statistically significant lesser post-operative pain in the initial 24 hours. The incidences of surgical site infections and port site herniation were lower in the umbilical port group. A large multicentric study may provide further insight into the preferred port site for the extraction of the gallbladder after laparoscopic cholecystectomy.
